# Supplementary material for: Evaluating accurate and efficient sampling strategies designed to measure social behavior and brush use in drylot housed cattle
Source: PLoS One. 2023 Jan 3;18(1):e0278233. doi: 10.1371/journal.pone.0278233 (PMC9810158; doi:10.1371/journal.pone.0278233)
Supplement: Attachment — Submitted filename: Response to Reviewers PONE-D-22-01864.docx [file pone.0278233.s002.docx]

September 26, 2022

Dr. Nei Moreira

Editor

PLOS ONE

Re: Manuscript ID PONE-D-22-01864

Dear Dr. Moreira and anonymous reviewers,

Thank you for the thorough and thoughtful review of our Manuscript ID PONE-D-22-01864 “Evaluating accurate and efficient sampling strategies designed to measure social behavior and brush use in drylot housed cattle”.

The suggestions made by the reviewers were very helpful to accomplish a more robust and clear paper for the reader. We are grateful for your input and recommendations. Should any part of this manuscript require further alteration, please let us know and we will make the necessary changes.

Below are our responses to the concerns raised by the anonymous reviewers.

Should you have any questions or concerns, please do not hesitate to contact us directly.

Sincerely,

Courtney L. Daigle, Ph.D.

Animal Behavior and Welfare Laboratory

Department of Animal Science

Texas A&M University

College Station, Texas

Reviewer #1: Many of the title words are found as keywords. Should you choose one or the other?

Evaluating accurate and efficient sampling strategies designed to measure social behavior and brush use in drylot housed cattle

**Response: Thank you for your feedback. New key words were selected, such as environmental enrichment, cattle brush, beef cattle, behavior, and stereotypies.**

well, which of the two goals is worth?
Thus, the objective of this study was to identify a sampling strategy that could accurately and efficiently record the performance of social, agonistic, and stereotypic behaviors in drylot-housed cattle with access to brushes.


DISCUSSION
The objective of this study was to determine which sampling strategies could accurately capture social and stereotypic behavior in cattle housed in feedlots with access to a brush.


The methodology was previously outlined in Park et al. (2020) ===?? In which of the 2020 works from? There are 3 of them! Didn't show up with a, b, c???

Park, R. M., Cramer, M. C., Wagner, B. K., Turner, P., Moraes, L. E., Viscardi, A. V., ... & Pairis-Garcia, M. D. (2020). A comparison of behavioural methodologies utilised to quantify deviations in piglet behaviour associated with castration. Animal Welfare, 29(3), 285-292.

Park, R. M., Schubach, K. M., Cooke, R. F., Herring, A. D., Jennings, J. S., & Daigle, C. L. (2020). Impact of a cattle brush on feedlot steer behavior, productivity and stress physiology. Applied Animal Behaviour Science, 228, 104995. <https://doi.org/10.1016/j.applanim.2020.104995>

Park, Rachel M., et al. "Impact of a cattle brush on feedlot steer behavior, productivity and stress physiology." Applied Animal Behaviour Science 228 (2020): 104995.

**Response: I am sorry for the confusion. We have made the corrections and updated the references.**

I think that the work was developed in North America, in the period from December 2017 to June 2018. It needed to have presented the average temperature at which the data set was collected. I needed the rain information. The average gain of the animals and correlated with behavior; temperature; rainfall. The work as it was presented, contributes little to the production system. Even if it's animal welfare.

**Response: A figure with THI, and average precipitations (in) per month in Bushland, TX from November 2017 to July 2018 was added. The goal of this specific paper was not to answer a biological question, per se. The point of this paper was to identify and evaluate different sampling strategies that can be used to collect the social, stereotypic, and environmental enrichment behaviors that would be of interest for a future study.**

Reviewer #2: o artigo tem mérito científico, esta bem redigido. Tem dados sólidos e coerentes e pode ser publicado. o material e métodos esta bem descrito. Os resultados obtidos são coerentes, e foram bem discutidos.

**Response: Obrigada pela análise e pelos comentários sobre meu artigo.**
